# Understanding challenges and barriers to quality end-of-life care for patients with hematologic malignancies: a GIMEMA survey

**DOI:** 10.1007/s00277-025-06594-6

**Published:** 2025-10-10

**Authors:** Leonardo Potenza, Fabio Efficace, Eleonora Borelli, Paola Fazi, Thomas Baldi, Francesca Tartaglia, Francesco Sparano, Claudio Cartoni, Pasquale Niscola, Claudia Mucciarini, Oreofe Odejide, Eduardo Bruera, Camilla Zimmermann, Marco Vignetti, Mario Luppi, Elena Bandieri

**Affiliations:** 1 Department of Medical and Surgical Sciences, University of Modena and Reggio Emilia, Modena, Italy; 2 Hematology Unit and Chair, Azienda Ospedaliera Universitaria di Modena, Modena, Italy; 3 Data Center and Health Outcomes Research Unit, Italian Group for Adult Hematologic Diseases (GIMEMA), Rome, Italy; 4 Hematology Unit, Department of Translational and Precision Medicine, Sapienza University, AOU Policlinico Umberto I, Rome, Italy; 5 Hematology Unit, Sant’Eugenio Hospital, Rome, Italy; 6 Medical Oncology Unit, AUSL Modena, Ramazzini Hospital, Carpi, Modena, Italy; 7 Department of Medical Oncology, Dana-Farber Cancer Institute, Boston, MA USA; 8 Palliative Care & Rehabilitation Medicine, UT MD Anderson Cancer Center, Houston, TX USA; 9 Department of Supportive Care, Princess Margaret Cancer Centre, University Health Network, Toronto, ON Canada; 10 Department of Medicine, University of Toronto, Toronto, ON Canada; 11 Oncology and Palliative Care Units, Civil Hospital Carpi, Local Health Agency (USL), Modena, Italy

**Keywords:** End-of-life care, Hematologic malignancies, Patient care planning, Palliative care, Survey

## Abstract

Patients with hematologic malignancies often receive aggressive end-of-life (EOL) care, which may be partly related to hematologists’ discomfort with discontinuing aggressive treatments at EOL. It is therefore important to investigate how hematologists perceive EOL care and how this affects their clinical practice. We assessed a cohort of Italian hematological oncologists through a GIMEMA online survey to explore their attitudes toward standard measures of quality EOL care, their opinions on barriers to providing this care, and potential interventions. EOL quality measures were defined acceptable to hematologist if at least 55% of respondents agreed with their suitability. One-hundred eight-six hematologists completed the survey. Hematologists rated 8 of 13 EOL quality measures as highly acceptable, including no new chemotherapy, no intensive care unit admission, no intubation/cardiopulmonary resuscitation in the last 30 days of life, and hospice admission > 7 days before death. Major barriers to quality EOL care included unrealistic patient expectations, clinician concerns about taking away hope, and uncertainty about what to say. Moreover, 73% admitted to being unfamiliar with discussing goals of care (GOC) or advance care planning (ACP). Suggested interventions for improvement included increasing the availability and timely integration of palliative care, and access to home care services. In conclusion, Italian hematologists find most standard EOL quality measures acceptable, they identify barriers to quality care, and are open to interventions, including early integration of palliative care, to improve patients’ EOL care. However, they lack familiarity with GOC and ACP discussions, highlighting the need for communication skills training.

**Supplementary Information:**

The online version contains supplementary material available at 10.1007/s00277-025-06594-6.

## Introduction

Several international organizations have advocated for the use of specific measures to assess the quality of end-of-life (EOL) care in patients with cancer, with the aim of reducing the overuse of aggressive treatments at EOL [1, 2]. These measures were originally developed by Earle and colleagues, and laid the foundation for the assessment of EOL care [1, 2]. Seminal studies have validated these indicators of low-quality EOL care, including chemotherapy within the last 14 or 30 days of life, more than one emergency room (ER) visit or at least one intensive care unit (ICU) admission or cardiopulmonary resuscitation (CPR) in the last month of life, stays of less than 7 days, and dying in a hospital rather than at home or in a hospice [1–5].

Despite the development of these measures, patients with hematologic malignancies (HM) continue to receive more aggressive EOL care than those with solid tumors. For example, 10–50% of patients with HM receive chemotherapy in the last 14 and 30 days of life, 50–90% are admitted to the ICU or hospital in the last month of life, and almost 50% are not offered hospice admission [4–13]. Factors contributing to this aggressiveness of EOL care may include the inherent prognostic uncertainty of many HM, which blurs the line between curable and terminal stages, as well as attitudes and beliefs held by hematologists, such as a greater tendency to perceive disease progression as a personal failure, reduced comfort in discussing EOL issues, and a higher propensity to offer systemic treatment even in the absence of survival benefit, which are distinct from those of medical oncologists [14–19]. Thus, to avoid unnecessary aggressive care at EOL for patients with HM, and to ensure that they receive EOL care consistent with their values and preferences, it is essential to investigate how hematologists perceive EOL and EOL care, and how these perceptions affect their clinical practice [20, 21].

A recent survey assessing a large cohort of US hematologic oncologists [15] revealed that the majority of participants considered standard EOL quality care measures to be highly acceptable. Additionally, the survey identified patients’ and clinicians’ expectations and concerns as the highest-ranked barriers to providing quality EOL care, such as unrealistic patient expectations, clinician concern about taking away hope, and unrealistic clinician expectations. Nevertheless, subsequent observational studies demonstrated that the prevalence of aggressive treatments at EOL among patients with HM remained unchanged [22, 23]. The existing evidence on this topic in Italy is limited, comprising an earlier study that exclusively examined the perception of palliative care among nurses and a small number of hematologists [24, 25].

The present study reports findings from a survey conducted by the Italian Group for Adult Hematologic Diseases (GIMEMA) among Italian hematologists, with the aim of understanding their attitudes and beliefs regarding EOL care and their level of agreement with standard measures of quality EOL care.

## Methods

### Survey design and development

Twenty-eight relevant questions were developed across 7 domains of interest (‘Demographic and Professional Profile’, ‘EOL Care’, ‘Signposts of the EOL Phase’, ‘Acceptability of EOL Quality Measures’, ‘Inpatient and Home Care’, ‘Barriers to EOL quality of care and potential interventions’, ‘Specialist Support’). The questionnaire was designed through a synthesis of preliminary data and relevant literature and previously published survey instruments and its applicability to the Italian context was assessed [15, 26, 27]. More details on the tool and its development are reported as Supplementary Material 1.

### Procedures and study sample

An online version of the survey was developed using the REDCap web-data collection tool [28], hosted at the GIMEMA Data Center (Rome, Italy). An email invitation outlining the study’s purpose and including a link to the survey was sent from the GIMEMA Data Center to all its affiliated centers on August 3, 2023.

The GIMEMA Foundation includes a large network of 140 Italian hematology centers, for which detailed information on the internal composition and the number of affiliated hematologists is not available. Through this organization, all the Italian centers that are part of the GIMEMA group are able to offer patients the same therapeutic and diagnostic possibilities regardless of where they are treated.

The invitation email specified that eligible participants would be at least one hematologist per center practicing in Italy who provide direct patient care to adults with HM. Instructions explicitly indicated that the survey addressed exclusively hematologists and no other healthcare professionals (e.g., nurses) and that the study’s objective was to characterize perspectives of Italian hematologists regarding the administration of EOL quality care and palliative care needs in patients with hematological malignancies, according to the latest suggestions of the American Society of Hematology [29]. The survey took approximately 20 min to be completed. Non-respondent centers received a maximum of 5 reminder emails once every two weeks; the email invitation is available as Supplementary Material (Supplement 2). The survey closed on October 20, 2023.

According to our Institutional Review Board, ethical review and approval were not required for this study as it used aggregate data from one online survey directed to physicians.

### Statistical analysis

Quantitative variables are presented as mean ± standard deviation and/or median and interquartile range, while qualitative variables are presented as absolute and percentage frequencies. Only surveys with at least 50% of items completed were considered valid and were included in the analysis. Quality EOL care measures (section IV) were considered acceptable if there was at least 55% agreement among hematologic oncologists about acceptability [15]. For the analyses, the 5-point Likert scales on agreement were converted into a binary variable, combining ‘Strongly disagree’, ‘Disagree’, and ‘Neither agree nor disagree’ vs. ‘Agree’ and ‘Strongly agree’ [30]. Barriers to high-quality EOL care were ascertained according to the percentage of study participants who reported issues as sometimes, often, or always a barrier. Additional analyses are reported in Supplementary Materials (Supplement 3).

Statistical comparisons were carried out using Pearson’s Chi-Squared tests or Fisher’s Exact tests, depending on the number of observations. Statistical analyses were conducted using R software with a significance level set at *p* <.05.

## Results

Of the 225 hematologists who opened and started to complete the survey, 186 (82.7%) completed at least 50% of the items included in it (median 2/center, range 1–8). Those 186 hematologists were from 93 out of 140 (66%) GIMEMA centers. Of these 93 centers, 50%, 25% and 25% were from Northern, Central and Southern Italy, respectively. The non-responding centers represented 33% from Northern Italy, 22% from Central Italy, and 45% from Southern Italy. A statistically significant difference (*p* =.04) was observed between responding and non-responding centers in terms of geographical distribution.

### Demographic and professional profile (section I)

The sample consisted of 104 females (56%) and 82 males (44%), with a median age of 50 years (IQR: 40–59) and a median of 23 years of professional experience (IQR: 14–32). Of the total respondents, 139 (75%) were community hospital-affiliated and 46 (25%) academic hospital-affiliated. The most frequently encountered HM was acute leukemia, reported by 147 respondents (79%), followed by myelodysplastic syndrome (*n* = 118, 63%), chronic leukemia (109, 59%), lymphoma (*n* = 107, 58%), and myeloma (*n* = 90, 48%). A significant portion (*n* = 137, 74%) of respondents treated transplant patients. Role models were the primary source of learning for EOL care for 143 (77%) respondents, followed by trial and error in clinical practice (*n* = 62, 33%), conferences and lectures (*n* = 56, 30%), and rotations in palliative or hospice care (*n* = 13, 7%). For a complete overview of the demographic and professional profile, see Table 1.

**Table 1:** Demographic and professional profile of participating hematologic oncologists (*N* = 186)

		*N* (%)
Gender		
Female		104 (56)
Male		82 (44)
Age, years		
Mean (SD)		50 (11)
Median (IQR)		50 (40–59)
Missing		1
Time since medical school graduation, years*		
Mean (SD)		23 (11)
Median (IQR)		23 (14–32)
Missing		1
Median time since medical school graduation, years*		
≥ 23		96 (52)
< 23		89 (48)
Missing		1
Medical specialties^§^		
Hematology		186 (100)
Oncology		12 (6)
Internal medicine		7 (4)
Other		1 (1)
Affiliation		
Community hospital		139 (75)
Academic hospital		46 (25)
Hematologic malignancies treated^§^		
Acute leukemia		147 (79)
Myelodysplastic syndrome		118 (63)
Chronic leukemia		109 (59)
Lymphoma		107 (58)
Multiple myeloma		90 (48)
Other		50 (27)
Method of learning to provide EOL care^§^		
Role models		143 (77)
Trial and error in clinical practice		62 (33)
Conferences and lectures		56 (30)
Rotation on a palliative care or hospice service		13 (7)
Other		13 (7)

*SD* Standard Deviation, *IQR* Interquartile Range, *EOL* End of Life.

*Time since medical school graduation’ referred to the most recent degree obtained.

^§^More than one answer was allowed

### **EOL care (section II)**

**Fig. 1:**
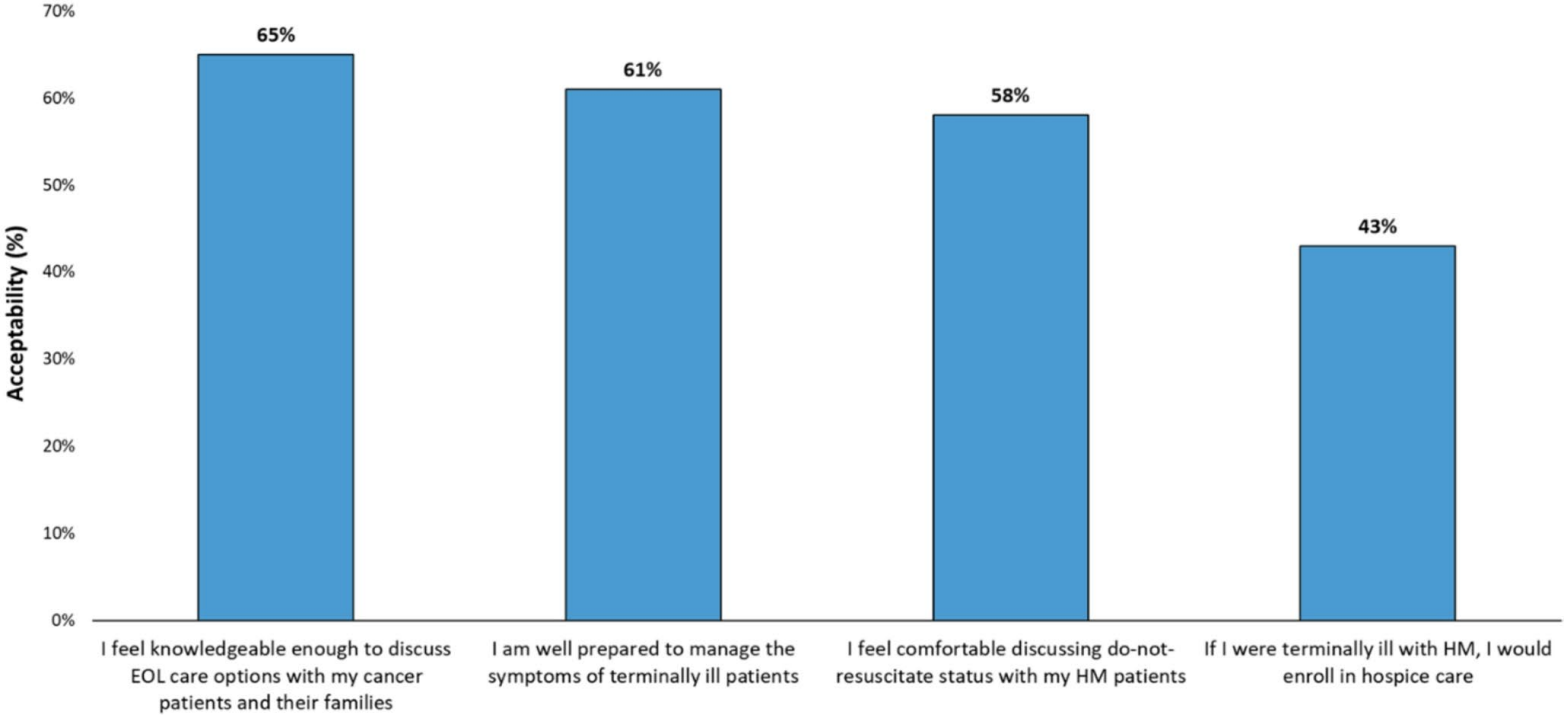
Participating hematologic oncologist’ perceptions of possessed competences in managing EOL (*N* = 186). Abbreviations: EOL = End of Life; HM = Hematologic Malignancies

A total of 117 respondents (63%) reported discussing prognosis with at least three-quarters of their patients. Moreover, 164 (88%) framed conversations around the potential curability of the disease, while only 76 (41%) framed them around potential incurability (more than one answer was allowed). Prognosis discussions typically occurred at diagnosis for 167 respondents (90%) and were revisited at relapse by 140 (75%). Goals of care and treatment priorities were discussed at diagnosis by 145 (78%) respondents. Regarding discussions about preferences for intubation or cardiopulmonary resuscitation (IOT/CPR) in case of sudden clinical deterioration, 70 (38%) addressed this at acute hospitalization, 40 (22%) at disease progression, and 61 (33%) when death was imminent. Hospice or home care services were discussed at progression by 94 (51%) and 112 (61%) respondents, respectively, and preferred place of death was addressed when death was imminent by 119 (66%). Additionally, 139 respondents (75%) felt that EOL discussions were initiated too late. Moreover, 74% (*n* = 136) admitted to being unfamiliar with the procedures for discussing GOC or defining an ACP [Supplement 4]. When asked about their confidence in providing EOL care, 114 (61%) agreed they were “well prepared to manage symptoms in terminally ill patients”, with males agreeing significantly more often than females (72% vs. 53%, *p* =.008). This gender gap in confidence was not explained by differences in seniority, as no statistically significant difference was found in the number of years since graduation between male and female respondents (median 26 years [IQR: 12–33] vs. 22 years [IQR: 14–30], respectively. One hundred and twenty respondents (65%) agreed they had “sufficient knowledge to discuss EOL care”, with males agreeing significantly more often than females (73% vs.58%, *p* =.035). When asked about comfort “discussing do-not-resuscitate status with my HM patients”, 109 (58%) agreed that they felt comfortable, with academic hospital-affiliated and males respondents significantly more likely to agree compared to respondents who were community hospital-affiliated and female (affiliation: 76% vs. 53%, *p* =.005; gender: 67% vs. 52%, *p* =.037). With respect to the kind of EOL care respondents would like to receive if they were terminally ill, 80 (43%) agreed they would opt for hospice care, with community hospital-affiliated respondents agreeing significantly more often than academic hospital-affiliated ones (48% vs. 26%, *p* =.009) [Figure 1]. There were no significant differences based on transplant patient management or years since graduation; results are not shown.

### Signposts of the EOL phase (section III)

When asked when they believed patients with various HM had a life expectancy of less than six months, respondents said that for acute myeloid leukemia or acute lymphoblastic leukemia, a guarded prognosis (< 6 months) was most likely at relapse after transplantation or cell therapy (43% and 54%, respectively) or at the first relapse (32% and 24%, respectively) [Supplementary 5]. In cases of Burkitt lymphoma, first relapse was the signpost for 43% of respondents, while relapse after transplantation was noted for diffuse large B-cell lymphoma (53%) and mantle cell lymphoma (49%). Conversely, a guarded prognosis for follicular lymphoma, chronic lymphocytic leukemia, and multiple myeloma typically followed third- or fourth-line treatment (47%, 45%, and 56%, respectively). Most respondents (48%) defined the EOL phase in HM as life expectancy < 3 months, with 39% considering it < 6 months [Supplement 5]. No significant differences were found regarding gender, affiliation, transplant patient management, or years since graduation.

### **Acceptability of EOL quality measures (section IV)**

**Fig. 2:**
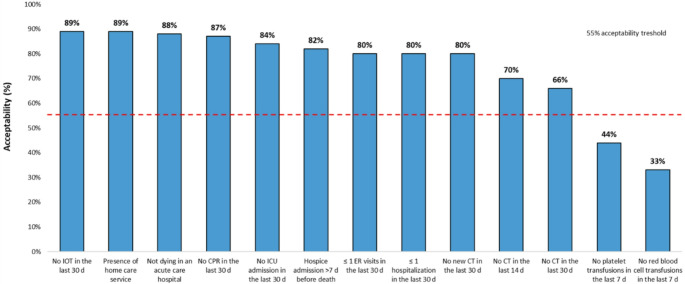
Acceptability of quality of EOL care measures as rated by Italian hematologic oncologists (*N* = 186). Abbreviations: CT = Chemotherapy; d = days; ICU = Intensive Care Unit; ER = Emergency Room; IOT = Intubation; CPR = Cardiopulmonary resuscitation

There was more than 55% agreement on all but two EOL quality measures: no red blood cell transfusions in the last seven days of life (33%), no platelet transfusions in the last seven days of life (44%) [Figure 2]. Nine standard EOL quality measures had over 75% agreement: no new chemotherapy in the last 30 days of life (80%), no intensive ICU admission in the last 30 days of life (84%), no IOT in the last 30 days of life (89%), and no CPR in the last 30 days of life (87%), no more than one ER visit in the last 30 days of life (80%), no more than one hospitalization in the last 30 days of life (80%), hospice admission > 7 days before death (82%), no death in an acute care hospital (88%), and presence of home care service (89%). Respondents with ≥ 23 years since medical school graduation were more likely to consider no chemotherapy in the last 14 days of life an acceptable indicator than respondents with < 23 years since graduating (78% vs. 64%, *p* =.037). To further explore this association, an additional analysis was conducted using quartile-based categories of years since graduation (4–13, 14–23, 24–32, 33–46 years). The findings confirmed a significant trend (*p* =.019), with the highest agreement (89%) observed among respondents with the longest professional experience (33–46 years). There were no significant differences in acceptability based on gender, affiliation, and provision of care for hematopoietic stem cell transplantation patients.

### **Hospice and home care services (section V)**

A total of 157 (85%) respondents agreed/strongly agreed that inpatient hospice was useful, while 74 (40%) felt that home care services were not appropriate for the level of care their patients required at EOL. However, 93 (50%) disagreed/strongly disagreed that inpatient hospice should be preferred to home care services. Only 62 (34%) said they would have referred more patients to hospice if they could visit them more often [Supplementary 6].

### **Barriers to high-quality EOL care and potential interventions (section VI)**

**Fig. 3:**
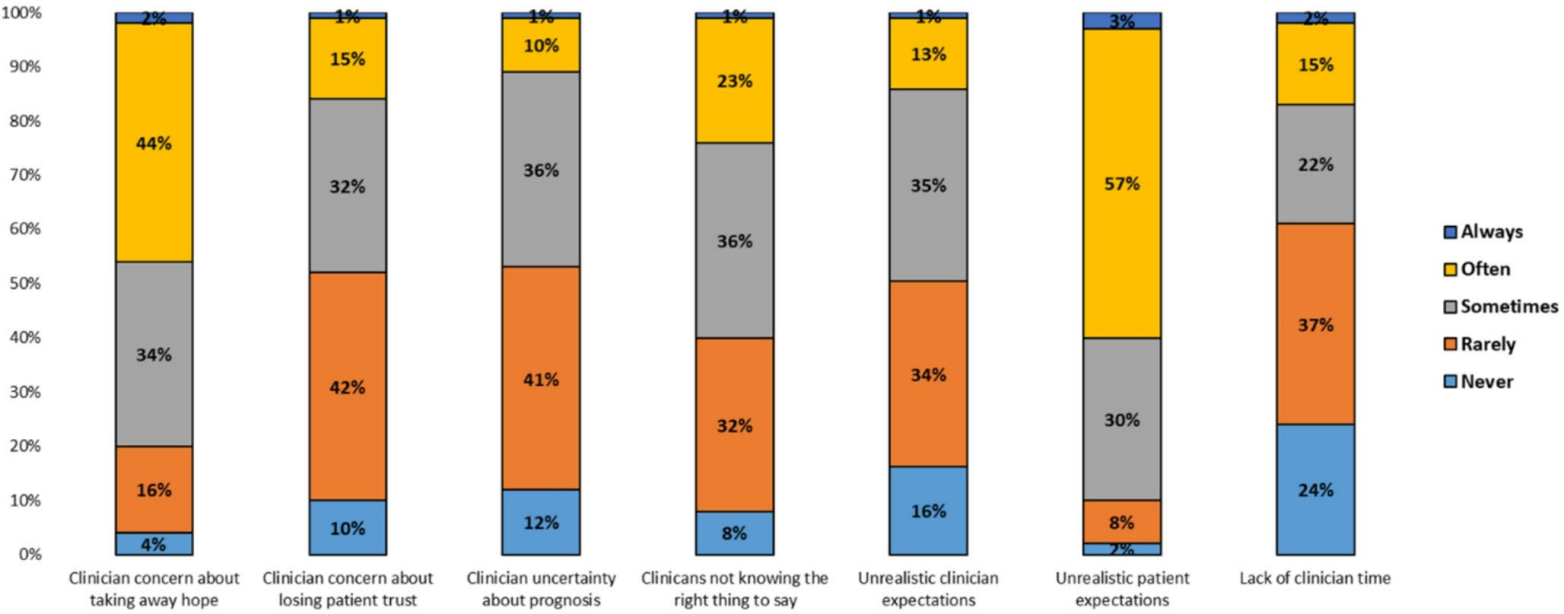
Perceived barriers to delivering quality EOL care as assessed by Italian hematologic oncologists (*N* = 186). Abbreviations: EOL = End of Life

The top three barriers reported by study participants were unrealistic patient expectations (90%), clinician concern about taking away hope (80%), and clinician not knowing the right thing to say (60%) [Figure 3]. The other barriers were perceived by less than 50% as sometimes, often, or always occurring. The highest ranked interventions perceived to be somewhat or very helpful in improving EOL care for HM patients were increasing access to palliative care services (100%), increasing access to home care services (99%), and early palliative care (96%) [Table 2].

**Table 2:** Data on potential interventions (*N* = 186)

	Not at all helpful	Somewhat helpful	Very helpful	Missing
A monthly tumor board to discuss issues relating to EOL care, n (%)	11 (6%)	107 (59%)	64 (35%)	4
Increasing access to inpatient hospice facilities, n (%)	12 (7%)	82 (45%)	89 (48%)	3
Increasing delivery of home care, n (%)	1 (1%)	28 (15%)	153 (84%)	4
Policies allowing hospice enrolment while still receiving disease-directed treatment, n (%)	16 (9%)	74 (40%)	93 (51%)	3
Increasing access to palliative care services, n (%)	0 (0%)	36 (20%)	147 (80%)	3
Integration of palliative care services with oncologic care at time of diagnosis, n (%)	7 (4%)	55 (30%)	120 (66%)	4
Prompts in the electronic medical record to encourage EOL care discussions (e.g. prompt when writing third-line chemotherapy), n (%)	58 (32%)	87 (47%)	39 (21%)	2

*EOL* End of Life

### **Specialist support (section VII).**

The highest ranked types of support by specialists, other than hematologists, that respondents would consider helpful, were: help managing relationships with local healthcare facilities (97%), help managing symptoms (92%), and helping patients adjust to the course of the disease (92%). The lowest ranked type of specialist support was help with explaining the prognosis to the patients and their families (54%) [Supplement 7].

## Discussion

In this national survey of Italian hematologic oncologists, the majority of standard EOL quality care measures were considered highly acceptable. The primary obstacles to delivering quality EOL care were identified by hematologists as unrealistic patient expectations, clinicians’ concern about taking away hope, and clinicians not knowing the right thing to say. The acknowledgement of not knowing the right thing to say was further corroborated by the high prevalence of unfamiliarity with GOC and ACP conversations. Potential interventions considered most useful for improving EOL care for HM patients included increasing the availability of palliative care services, particularly through early integration, and enhancing access to home care services. Interestingly, this widespread unfamiliarity with GOC and ACP procedures coexists with a relatively high self-reported frequency of conversations about prognosis and treatment priorities. This apparent inconsistency likely reflects a lack of awareness about the existence of structured models and evidence-based procedures for EOL communication. In practice, many hematologists may conduct these discussions in an intuitive and unstructured way, without formal training or reference to recognized frameworks and most likely perform these conversations as a one-time communication without a series of follow-up meetings that facilitate the cultivation of prognostic awareness and enable patients to receive care that aligns with their goals and preferences [31]. This is further supported by our finding that “help with explaining the prognosis” was ranked lowest among the preferred forms of specialist support. This apparent paradox highlights a significant knowledge gap, whereby clinicians may not recognize the added value of specialist guidance due to limited exposure to structured communication models. As suggested by recent evidence [13], the adoption of serious illness communication frameworks in hematology could enhance the consistency and quality of prognosis and GOC discussions, ultimately fostering more effective early integration of palliative care.

More than half of EOL quality measures were considered by most respondents to be highly acceptable, and over two-thirds were deemed acceptable by more than 65% of hematologists. In contrast, the two measures related to restricting transfusions in the last week of life were deemed unacceptable by over half of the respondents. This suggests that hematologic oncologists may view transfusions near the EOL to be more palliative and less reflective of aggressive care. No differences were observed between the acceptability rates of measures that were physician-dependent (e.g., ≤ 1 hospitalization in the last 30 days) versus patient-dependent (e.g., ≤ 1 ER visit in the last 30 days). These results are in keeping with those observed among US hematologic oncologists [15]. In contrast to the latter study, which included hematologic oncologists who also treated patients with solid tumors, our data were obtained from a homogeneous sample of physicians exclusively managing HM, including those practicing in tertiary care centers offering hematopoietic stem cell transplantation and experimental therapies. Taken together, our analysis highlights the need to develop interventions to improve adherence to existing, established, and widely accepted measures, rather than focusing on identifying new ones.

Notably, the presence of home care services was deemed acceptable by almost 9 out of 10 respondents. In fact, it is well known that one of the main providers of hematological home care services in Italy is the Associazione Italiana contro le Leucemie, Linfomi e Mieloma (AIL). However, of 83 AIL sections throughout the country, only 33 provide a home care service [32]. This suggests that there is a need to increase home care services across Italy and challenges hematologists’ skepticism about managing HM patients at home during the EOL phase [33–36]. Despite the high acceptability of home care services, more than a third of respondents felt that home care services were inadequate for the level of care required by their patients. This tension between acceptability and adequacy of services may be due to hematologic oncologists’ concerns that the symptom burden of their patients, particularly with respect to anemia, thrombocytopenia, and the need for blood products, are not fully addressed with home care services alone [14, 24, 37]. We thus need research to identify and develop innovative ways to bolster home care services so that they are well tailored to the unique needs of patients with blood cancers.

In alignment with the findings of studies in United States, France, and Belgium, our cohort also demonstrated that concerns about the potential impact of discussing EOL care on the patient-physician relationship remain significant [14, 21, 24, 37]. Indeed, more than three-quarters of Italian hematologists consider the concern of taking away patients’ hope to be a barrier to providing quality EOL care. Although, the most frequently reported obstacle was unrealistic patient expectations, it is important to acknowledge that these expectations are often shaped by the way doctors communicate [14]. Consistent with this, the third most frequently identified barrier was clinicians not knowing the right thing to say.

Hematologists’ stated discomfort with communication at EOL aligns with the finding that most hematologists were unfamiliar with procedures for both GOC conversations and ACP. Additionally, around two-fifths of respondents reported not feeling adequately prepared to manage EOL symptoms and not feeling comfortable or knowledgeable enough to handle and discuss EOL care. These findings are consistent with existing literature of higher levels of discomfort with discussing death and dying among hematology specialists [14]. These data underscore the need for effective communication to enhance patient trust and perceived quality of care in EOL settings, and strongly support the need for communication skills training for Italian hematologists [38]. Consistently, responses to the item regarding the presence of role models in EOL care further highlight the lack of structured training: only a small minority of participants (6.7%) reported referring to a palliative care specialist as a role model during their education. Similarly, the most frequently reported sources of learning were informal, such as clinical experience and peer discussion, rather than formal postgraduate training. These findings align with previous research in the Italian context, which identified a lack of structured teaching on palliative care and highlighted anticipatory discomfort among medical students when dealing with death and dying, underscoring the need for early and integrated palliative care education [39]. Until the academic year 2021–2022, Italy had no national Specialty School in Palliative Medicine, and palliative care was not systematically included in hematology training programs, which may have contributed to enduring misconceptions equating palliative care with terminal care only [40]. Of further interest, hematology was neither included among the specific minimum structural requirements for the suitability of the training clinical network type nor among the disciplinary scientific sectors, considered compulsory and indispensable, in the new Italian School in Medicine and Palliative Care, unlike internal medicine, medical oncology, neurology, anesthesia, intensive care, and others [41].

Notably, seniority appeared to influence attitudes and confidence regarding EOL care. Hematologists with more years since graduation were significantly more likely to consider “no chemotherapy in the last 14 days of life” as an acceptable quality indicator. This association remained significant when using quartile-based categories, suggesting that both generational differences in training and accumulated clinical experience may shape clinicians’ views on aggressive treatments at the EOL.

It has been shown that such training and communication of quality-improvement interventions are associated with significant increases in physicians’ skills [38, 42]. These interventions can help to improve the frequency and quality of documented EOL conversations, and more importantly, can increase the likelihood that patients receive EOL care that is aligned with their preferences [38, 43, 44]. These conversations become more comprehensive and patient-centered, focusing on values, goals, illness understanding, and preferences for life-sustaining treatments [43, 44].

Regarding interventions to improve the quality of EOL care for their patients, most of our cohort emphasized the importance of increasing access to palliative care services, particularly through early integration, as well as enhancing home care services. Studies have indicated that early integration of palliative care for patients with HM can lead to several benefits, including improved quality of life and quality of care and reduced aggressiveness at the EOL [45–47]. EPC may also help to align patient and clinician expectations and support realistic hope [48]. Additionally, palliative home care programs are associated with lower rates of infections and weekly transfusions compared to the hospital setting [49]. A recent Italian study, including nurses and hematologists, showed that despite a substantial understanding of the significance and advantages of palliative care, there was a significant perceived variability in the role of simultaneous care and in the optimal timing for palliative care referral, often delaying them until the patient’s prognosis is less than three months or symptoms become unmanageable, due to uncertain disease trajectories and cultural-organizational barriers that emphasize curative treatment over EPC integration [25]. Our study in a homogeneous sample of hematologists demonstrated greater awareness of benefits of early integration of palliative care. Therefore, further efforts may be required to extend information regarding this model of care to nursing staff, who are integral to its implementation.

Approximately two-thirds of respondents indicated that the onset of the EOL phase occurred at relapse after first-line treatment or transplantation/cell therapy for acute leukemia, and after transplantation/cell therapy and during the third or fourth line of treatment for aggressive lymphomas and multiple myeloma. Conversely, there was greater heterogeneity and a lack of consensus when applying this definition to follicular lymphoma or chronic lymphocytic leukemia. These latter results, combined with the median age of incidence for indolent or chronic HM, suggest that new signposts for the EOL phase should be evaluated for these patients. This is crucial to avoid the risk of aggressive interventions at the EOL, especially considering that hematologic specialists are more likely to prescribe systemic therapies with moderate toxicity and no survival benefit for patients with poor performance status and limited life expectancy [14]. Identification of clearer signposts can also be used as triggers for readdressing EOL discussions, to avoid initiating these discussions too late in the course of the disease [26].

Our study has several limitations. First, given the large number of centers involved, suggesting that a significant proportion of hematologists did not respond, and the uneven geographical representation among responding and non-responding centers, the generalizability of our findings may not be optimal. Second, there is potential for selection bias, as respondents may have been more inclined than non-respondents to engage with topics covered in the survey. Third, it should be noted that the survey is based on measures adapted from a potentially different cultural context in the US. Consequently, the results may not be as fully representative of Italian perspectives, as if they had been identified by local, larger focus groups, including physicians and other stakeholders such as family caregivers and patient advocacy groups. However, the high rates of acceptability observed in our study regarding these measures may mitigate this concern. Fourth, although we collected data on whether respondents were affiliated with community or academic hospitals, we do not have information on the actual distribution of EOL care delivery across these settings in Italy. Therefore, it is not possible to determine whether the high proportion of respondents from community hospitals reflects the settings where most EOL care for hematology patients is actually provided nor whether the level of care impacts willingness to provide EOL care.

In conclusion, our findings indicate that a substantial proportion of Italian hematologists find most current EOL quality measures to be acceptable, can identify specific barriers to achieving quality care, and are receptive to interventions for its improvement. However, the stated discomfort with EOL conversations and the high level of unfamiliarity with procedures for GOC or ACP conversations highlights the urgent need for targeted communication skills training for hematologists. Finally, given that early involvement of palliative care services was the most highly rated intervention to enhance EOL quality for patients with HM, our data strongly support timely integration of palliative care for these patients.

## Supplementary Information

Below is the link to the electronic supplementary material.

Supplementary Material 1 (DOCX 37.9 KB) 

## Data Availability

The data that support the findings of this study are not openly available due to reasons of sensitivity and are available from the corresponding author upon reasonable request.
