# Supplementary material for: Understanding challenges and barriers to quality end-of-life care for patients with hematologic malignancies: a GIMEMA survey
Source: Ann Hematol. 2025 Oct 10;104(10):5423–32. doi: 10.1007/s00277-025-06594-6 (PMC12619704; doi:10.1007/s00277-025-06594-6)
Supplement: Supplementary file 1 — Supplementary Material 1 (DOCX 37.9 KB) [file 277_2025_6594_MOESM1_ESM.docx]

**Supplementary Material 1**. ***Survey Design and Development***

The applicability of the identified questions to the Italian context was assessed through four focus groups between two hematologic oncologists (LP, ML), one oncologist (EBa), one clinical psychologists (FE), and one psycholinguist (EBo).

Section I (‘Demographic and Professional Profile’) aimed to characterize the sample from a demographic and professional perspective; section II (‘EOL Care’) aimed to determine how respondents define and manage EOL; section III (‘Signposts of the EOL Phase’) aimed to investigate their knowledge on the implementation of goals of care (GOC) and advance care plan (ACP) conversations; section IV (‘Indicators of EOL Quality of Care’) aimed to explore perspectives on quality measures of care near EOL. In addition to indicators extracted from a similar survey,^10^ three new specific measures were added: ‘No chemotherapy in the last 30 days of life’, ‘No new chemotherapy in the last 30 days of life’, and ‘No provision of home care services’. Respondents were asked to rate each measure as ‘Acceptable’ or ‘Not acceptable’. Section V (‘Inpatient and Home Care’) aimed to explore respondents' perceptions of inpatient and home care services for HM patients at EOL. Respondents were presented with a series of statements to evaluate on a 5-point Likert scale ranging from ‘Strongly disagree’ to ‘Strongly agree’. Section VI (‘Barriers to EOL Quality of Care and Potential Interventions’) aimed to identify physician- and patient-related barriers that may hinder the quality of EOL care. Barriers’ frequency was ranked on a 5-point Likert scale ranging from ‘Never’ to ‘Always’. Additionally, respondents were asked to provide opinions on interventions that could improve EOL care, ranked on a 3-point Likert scale ranging from ‘Not at all useful’ to ‘Extremely useful’. Section VII (‘Specialist Support’, 2 items) aimed to assess the perceived usefulness of specialized support interventions to hematologic clinical practice in improving EOL care and was new compared to previously published surveys on the same topic. These interventions were ranked on a 5-point Likert scale ranging from ‘Not at all helpful’ to ‘Extremely helpful’.

**Supplementary Material 2.** Email invitation.

**ITALIAN TEXT**

Gentile Dottoressa/Dottore,

Siamo lieti di proporle la partecipazione ad una Survey della Fondazione GIMEMA sul tema delle cure palliative nei pazienti ematologici. L’obiettivo generale di questa Survey, che nasce anche dall’ultimo Meeting organizzato dalla Fondazione GIMEMA lo scorso 19 Maggio 2023 a Roma, è quello comprendere meglio l’atteggiamento dei medici ematologi italiani rispetto ai bisogni di cure palliative in ematologia. Negli ultimi anni, una maggiore attenzione è stata posta dalla comunità ematologica internazionale su questo tema di ricerca come indicato, ad esempio, dall’istituzione di sessioni di formazione presso l’American Society of Hematology (ASH) Meeting (Odejide OO. Strategies for introducing palliative care in the management of relapsed or refractory aggressive lymphomas. Hematology Am Soc Hematol Educ Program. 2020 Dec 4;2020(1):148-153).

I nuovi dati raccolti da questo survey potrebbero essere la base di una pubblicazione scientifica ed il punto di partenza per esperienze di ricerca condivise e di documenti di riflessione e di consenso sul tema delle cure palliative, incluse le cure palliative precoci, in emato-oncologia. Al termine della survey, dovrà cliccare il tasto ‘submit’ per inviare le sue risposte. Per iniziare la survey, può cliccare il seguente link o copiarlo nel suo browser: LINK.

La ringraziamo per la Sua attenzione e collaborazione

Cordiali saluti.

**ENGLISH TEXT**

Dear Doctor,

We are pleased to invite you to participate in a survey by the GIMEMA Foundation on the topic of palliative care in hematologic patients. The general aim of this survey, which also stems from the last meeting organized by the GIMEMA Foundation on May 19, 2023, in Rome, is to better understand the attitudes of Italian hematologists regarding the palliative care needs in hematology. In recent years, the international hematology community has placed greater emphasis on this area of research, as indicated, for example, by the establishment of training sessions at the American Society of Hematology (ASH) Meeting (Odejide OO. Strategies for introducing palliative care in the management of relapsed or refractory aggressive lymphomas. Hematology Am Soc Hematol Educ Program. 2020 Dec 4;2020(1):148-153).

The new data collected from this survey could serve as the basis for a scientific publication and as a starting point for shared research experiences and the development of reflective and consensus documents on the topic of palliative care, including early palliative care, in onco-hematology.

At the end of the survey, you will need to click the ‘submit’ button to send your responses. To start the survey, you can click on the following link or copy it into your browser: LINK.

We thank you for your attention and collaboration.

Best regards.

**Supplementary Material 3.** Additional Analyses.

Additional analyses were conducted to profile respondents who provided unexpected or distinctive answers. Specifically, we profiled respondents based on their agreement or disagreement with EOL care statements ‘I am well prepared to manage the symptoms of terminally ill patients’, ‘I feel knowledgeable enough to discuss EOL care options with my cancer patients and their families’, ‘I feel comfortable discussing do-not-resuscitate status with my cancer patients’, and ‘If I were terminally ill with cancer, I would enroll in hospice’ (Section II); respondents who indicated ‘Less than 3 months’ in response to the question “Which of the following life expectancies would you define as end-of-life in cases of hematologic malignancies?” (Section III); and respondents who defined acceptable or not acceptable as quality EOL care measure ‘No chemotherapy in the last 14 days of life’ and ‘No chemotherapy in the last 30 days of life’ (Section IV). Profiling was conducted based on respondents' gender (male vs. female), type of affiliation (hospital-based vs. academic), management of transplant patients (yes vs. no), and years since graduation (<23 vs. ≥23).

**Supplementary Material 4.** Data on EOL care (N=186).

|  |  | |  | **N (%)** |
| --- | --- | --- | --- | --- |
| **With what percentage of your patients with HM do you discuss prognosis?** | | | |  |
|  | 76 to 95% | |  | 63 (34) |
|  | more than 95% | |  | 54 (29) |
|  | 51 to 75% | |  | 39 (21) |
|  | 26 to 50% | |  | 24 (13) |
|  | less than or equal to 25% | |  | 6 (3) |
| **What is your preferred terminology for discussing prognosis with patients? *** | | | |  |
|  | General discussion of whether the disease is potentially curable | |  | 164 (88) |
|  | General discussion of whether the disease is potentially incurable | |  | 76 (41) |
|  | Percentage of survival | |  | 57 (31) |
|  | Median survival (months or years) | |  | 53 (28) |
|  | Percentage of mortality | |  | 25 (13) |
|  | Median mortality (months or years) | |  | 8 (4) |
| **When do you typically conduct the initial discussion addressing prognosis with your patients?** | | | |  |
|  | Upon presentation or diagnosis | |  | 167 (90) |
|  | At the relapse | |  | 8 (4) |
|  | During a period of stability | |  | 5 (3) |
|  | During an acute phase of illness | |  | 3 (1.5) |
|  | When death is clearly imminent | |  | 3 (1.5) |
| **At what point during the course of disease do you re-address prognosis with your patients?** | | | |  |
|  | At the relapse | |  | 140 (75) |
|  | During an acute phase of illness | |  | 30 (16) |
|  | During a period of stability | |  | 8 (4) |
|  | When death is clearly imminent | |  | 7 (4) |
|  | In general, I do not re-address prognosis after the initial discussion | |  | 1 (1) |
| **For patients with life-threatening HM, when do you typically conduct the initial discussion specifically addressing:** | | | |  |
|  | | **Broad goals for medical care and treatment priorities?** | Upon presentation or diagnosis | 145 (78) |
|  |  |  | During a period of stability | 1 (1) |
|  |  |  | During an acute hospitalization | 7 (4) |
|  |  |  | Upon disease progression | 32 (17) |
|  |  |  | When death is clearly imminent | 1 (1) |
|  |  |  | Missing | 0 |
|  | | **Preferences for cardiopulmonary resuscitation** **and/or intubation in case of a sudden deterioration in clinical condition?** | Upon presentation or diagnosis | 12 (6) |
|  |  |  | During a period of stability | 2 (1) |
|  |  |  | During an acute hospitalization | 70 (38) |
|  |  |  | Upon disease progression | 40 (22) |
|  |  |  | When death is clearly imminent | 61 (33) |
|  |  |  | Missing | 1 |
|  | | **Preferred site of death?** | Upon presentation or diagnosis | 2 (1) |
|  |  |  | During a period of stability | 2 (1) |
|  |  |  | During an acute hospitalization | 8 (4) |
|  |  |  | Upon disease progression | 49 (27) |
|  |  |  | When death is clearly imminent | 119 (66) |
|  |  |  | Missing | 6 |
|  | | **Inpatient hospice enrollment?** | Upon presentation or diagnosis | 3 (2) |
|  |  |  | During a period of stability | 1 (1) |
|  |  |  | During an acute hospitalization | 12 (7) |
|  |  |  | Upon disease progression | 94 (51) |
|  |  |  | When death is clearly imminent | 74 (40) |
|  |  |  | Missing | 2 |
|  | | **Home care service enrollment** | Upon presentation or diagnosis | 14 (8) |
|  |  |  | During a period of stability | 7 (4) |
|  |  |  | During an acute hospitalization | 25 (14) |
|  |  |  | Upon disease progression | 112 (61) |
|  |  |  | When death is clearly imminent | 26 (14) |
|  |  |  | Missing | 2 |
| **In your experience, EOL care discussions with patients who have HM typically occur:** | | | |  |
|  | Too late | |  | 139 (75) |
|  | At the right time | |  | 43 (23) |
|  | Too early | |  | 3 (2) |
|  | Missing | |  | 1 |
| **Do you know the procedure to discuss ACP?** | | | |  |
|  | No | |  | 136 (74) |
|  | Yes | |  | 49 (26) |
|  | Missing | |  | 1 |
| **Do you know the procedure to discuss GOC?** | | | |  |
|  | No | |  | 136 (74) |
|  | Yes | |  | 49 (26) |
|  | Missing | |  | 1 |
| *More than one answer was allowed. HM = Hematologic Malignancies; EOL = End Of Life; GOC = Goals Of Care. | | | | |
|  | | | |  |

**Supplementary Material 5.** Italian hematologic oncologists’ perspectives regarding signposts of a life expectancy < 6 months for various HM, using a 60-year-old patient with disease evaluated as a reference (N=186).

|  | **Relapse after cell therapy (stem cell transplant/CAR-T)** | **Relapse after third- or fourth-line treatment** | **First relapse** | **Diagnosis** | **None of the previous because life expectancy is > 6 months in these scenarios** | **N/A – I don’t treat this disease** | **Missing** |
| --- | --- | --- | --- | --- | --- | --- | --- |
| Mantle cell lymphoma, n (%) | 90 (49%) | 57 (31%) | 8 (4%) | 0 | 3 (2%) | 25 (14%) | 3 |
| Diffuse large B-cell lymphoma, n (%) | 98 (53%) | 49 (27%) | 10 (5%) | 1 (1%) | 1 (1%) | 25 (14%) | 2 |
| Follicular lymphoma, n (%) | 15 (8%) | 85 (47%) | 1 (1%) | 0 | 52 (29%) | 28 (15%) | 6 |
| Burkitt lymphoma, n (%) | 46 (25%) | 30 (16%) | 78 (43%) | 7 (4%) | 1 (1%) | 21 (11%) | 3 |
| Chronic lymphocytic leukemia, n (%) | 2 (1%) | 81 (45%) | 0 | 0 | 71 (39%) | 26 (14%) | 6 |
| Acute Lymphoblastic Leukemia, n (%) | 99 (54%) | 15 (8%) | 45 (24%) | 9 (5%) | 1 (1%) | 16 (9%) | 1 |
| Acute myeloid leukemia, n (%) | 79 (43%) | 24 (13%) | 59 (32%) | 9 (5%) | 0 | 13 (7%) | 2 |
| Multiple myeloma, n (%) | 11 (6%) | 102 (56%) | 2 (1%) | 1 (1%) | 35 (19%) | 30 (17%) | 5 |

HM = Hematologic Malignancies; N/A = Not Applicable.

**Supplementary Material 6**. Data on hospice and home care service (N=186).

|  | **Strongly disagree** | **Disagree** | **Neutral** | **Agree** | **Strongly agree** | **Missing** | |
| --- | --- | --- | --- | --- | --- | --- | --- |
| Inpatient hospice is useful, n (%) | 2 (1%) | 11 (6%) | 14 (8%) | 101 (55%) | 56 (30%) | 2 | |
| I feel that home care service is not appropriate for the level of care that patients required, n (%) | 22 (12%) | 60 (33%) | 28 (15%) | 54 (29%) | 20 (11%) | 2 | |
| My patients feel that home care service is not appropriate for the level of care that they need, n (%) | 12 (7%) | 63 (35%) | 39 (21%) | 53 (29%) | 15 (8%) | 4 | |
| For my patients, I prefer inpatient hospice to home care services, n (%) | 26 (14%) | 67 (36%) | 63 (34%) | 21 (11%) | 7 (5%) | 2 | |
| I would refer more patients to hospice or home care service if I knew I could visit them more (more than once a month) , n (%) | 12 (7%) | 50 (27%) | 57 (31%) | 55 (30%) | 9 (5%) | 3 | |
|  | | | | | | |  |

**Supplementary Material 7**. Data on specialist support (N=186).

|  | **Not at all helpful** | **2** | **3** | **4** | **Strongly helpful** | **Missing** |
| --- | --- | --- | --- | --- | --- | --- |
| A specialist who supports you in managing the debilitating symptoms resulting from the disease and treatments, n (%) | 2 (1%) | 12 (7%) | 33 (18%) | 78 (42%) | 59 (32%) | 2 |
| A specialist who explains to the patient with you the possible and probable course of the disease trajectory, n (%) | 16 (9%) | 38 (21%) | 53 (29%) | 52 (28%) | 24 (13%) | 3 |
| A specialist who supports your patients in adapting to the course of the disease, n (%) | 2 (1%) | 12 (7%) | 50 (27%) | 65 (35%) | 55 (30%) | 2 |
| A specialist who explains the prognosis of the disease to your patients and their families, n (%) | 35 (19%) | 50 (27%) | 42 (23%) | 31 (17%) | 26 (14%) | 2 |
| A specialist who helps you manage the patient and their families when therapeutic options are limited, n (%) | 8 (4%) | 20 (11%) | 34 (19%) | 66 (36%) | 54 (30%) | 4 |
| A specialist who assists you in managing relationships with local healthcare facilities (home hospice, community care, hospital/community hospice), n (%) | 1 (1%) | 4 (2%) | 17 (9%) | 54 (30%) | 107 (58%) | 3 |
|  | | | | | |  |
